# *In Vitro* Comparison of Cone Beam Computed Tomography with Digital Periapical Radiography for Detection of Vertical Root Fracture in Posterior Teeth

**Published:** 2016-06

**Authors:** Mehrdad Abdinian, Hamid Razavian, Nastaran Jenabi

**Affiliations:** 1 Dental Implants Research Center, Dept. of Oral and Maxillofacial Radiology, School of Dentistry, Isfahan University of Medical Sciences, Isfahan, Iran.; 2 Torabinejad Dental Research Center, Dept. of Endodontics, School of Dentistry, Isfahan University of Medical Sciences, Isfahan, Iran.; 3 Postgraduate Student, Dept. of Orthodontics, Dental School, Isfahan (Khorasgan) Branch, Islamic Azad University, Isfahan, Iran.

**Keywords:** Cone-Beam Computerized Tomography, Dental Digital Radiography, Tooth Fracture, Diagnosis

## Abstract

**Statement of the Problem:**

The diagnosis of vertical root fracture (VRF) is a challenging task.

**Purpose:**

This *in vitro* study compared cone beam computed tomography (CBCT) imaging with digital periapical radiography (DPR) made by three different horizontal angels (20°mesial, 0° and 20° distal) for accurate diagnosis of VRF.

**Materials and Method:**

Among 120 posterior teeth included in this study, 60 were vertically fractured. Fractured and non-fractured teeth were randomly distributed into three groups defined as group 1 with no filling in the root canal, group 2 with gutta-percha in the canal, and group 3 with the intracanal post. All samples were placed in a dry mandible and imaged with CBCT and DPR techniques. Two blind observers investigated the images.

**Results:**

CBCT had higher sensitivity but lower specificity compared with DPR, except for the intracanal post group in which the sensitivity of DPR was higher; though the chi-square test showed the differences to be statistically insignificant. The sensitivity, specificity, and accuracy of CBCT and DPR were reduced in the cases that gutta-percha or post were present in the canal. Inter-observer agreement was higher for CBCT. A set of three DPRs with different horizontal angels were significantly more sensitive for VRF recognition than a single orthogonal DPR.

**Conclusion:**

Based on our results, there was no significant difference between CBCT and a set of three DPRs with different angulations for VRF detection in posterior teeth. Therefore, it is suggested to consider DPRs with three different horizontal angels (20°mesial, 0° and 20° distal) for radiographic evaluation before CBCT examination.

## Introduction

Vertical root fracture (VRF) is a longitudinal crack extended along the tooth root.[1] Previous root canal treatment and placement of a post, especially a short one, have been reported in most of the extracted teeth with VRF.[2-3]

The diagnosis of VRF is nearly always a challenging task. A series of clinical and radiographic signs along with the clues in the patient’s history and complaints suggest VRF in a tooth. But none of these signs are pathognomonic[4] and the diagnosis can only be confirmed by direct inspection of the fracture line in the suspected tooth.[1] Poor prognosis and progressive bone resorption leave no option but to extract the fractured tooth in most cases.[1] In order not to waste more cost and effort on further treatments and also to prevent more destruction in the surrounding tissues, it is important to differentiate VRF from other possible pathologic conditions.[5]

The radiolucency of the fracture line is one of the first radiographic signs.[1, 5-6] Most of the time, it is hardly detectable in periapical radiographs because the horizontal angle between the projected beam and the fracture plane must be less than 4 degrees.[6] In addition, superimpositions may prevent proper detection of the fracture line. In some reports, the cone beam computed tomography (CBCT) is considered superior to the periapical radiography (PR) for VRF recognition,[7-8] however; Kambungton *et al.*[9] found the difference to be insignificant. Furthermore, higher doses of radiation exposure and costs in comparison with intra-oral radiography[10] and occurrence of artifacts in the presence of radio-opaque filling materials may limit CBCT application.[11]

Da Silveria *et al.*[12] reported that the sensitivity and specificity of PR for VRF diagnosis in single-rooted teeth with opaque root filling material and post were improved by using different horizontal angles and this approach should be considered prior to CBCT application. Kambungton *et al.*[9] similarly concluded that PR examination with three different horizontal angles was more effective than a single orthogonal projection in single-rooted non-filled teeth. Despite several researches, it remains unclear whether it is prudent to prescribe CBCT for VRF diagnosis. VRF is more prevalent in posterior teeth. Studies focusing on posterior teeth might be more practical.[5] The purpose of this study was to compare the accuracy of CBCT and digital periapical radiography (DPR) taken at three different horizontal angles for detection of VRF in root filled and post cemented posterior teeth. 

## Materials and Method

This analytical cross-sectional study was approved by the Ethics Committee of Isfahan University of Medical Sciences.

**Sample preparation**

This study used 120 extracted intact human mandibular posterior teeth (60 premolars, 60 molars). After debridement with ultrasonic scaler (Ultrasonic Cleaner LX; Faraz Mehr Isfahan Co., Iran), root surfaces of the extracted teeth were stained with 1% methylene blue and inspected with a 5× magnifier. Teeth with any previous crack or fracture were excluded. 

After the preparation of a straight-line access, cleaning and shaping of the canals were completed with passive step-back technique by using #10 to 40 K-type files (Dentsply-Maillefer; Ballaigues, Switzerland) and #2 and 3 Gates Glidden drills (Dentsply-Maillefer; Switzerland). Forty teeth were selected randomly and the root canals of the 80 remaining teeth were obturated through the lateral condensation of #20 to 45 gutta-percha cones (Aria Dent; Tehran, Iran) and by using AH26 root canal sealer (Dentsply Tulsa Dental; Switzerland). After complete setting of the sealer, the gutta-percha of the premolar canals and one of the molar canals were removed by using #2 or 3 Peeso reamer drill (Dentsply-Maillefer; Switzerland) in a way that at least 4 mm of gutta-percha remained at the apical end. Half of the filled and unfilled teeth were randomly selected. Then, a custom made pin was inserted in the vacant space of the canal. By using a hammer, intermittent strokes were applied in a controlled manner until fracture occurred. The teeth with separated parts were excluded. The presence of fracture was confirmed by using staining method described above. The orientation of fracture line was recorded.  

The fractured teeth were randomly distributed into three groups. Samples in group 1 had no filling material in the canals, group 2 had a prefabricated screw-type post (Svenska Dentorama AB; Stockholm, Sweden) suitable for the canal in size and length which was cemented with zinc phosphate cement (Prime-dent; Chicago, USA), and in group 3, the canals were re-filled with gutta-percha (Aria Dent; Iran). Non-fractured teeth were randomly divided into three groups similar to the fractured teeth. All groups included an equal number of molars and premolars (10 molars and 10 premolars).

Finally, all the access cavities were filled with dental amalgam (Aristaloy; Birmingham, UK). In order to prevent intrusion of amalgam into the non-filled canal spaces during condensation, a small piece of cotton was placed in the canal orifice before filling the access cavity.

A dry human male mandible with relatively huge tooth sockets was selected. In order to adapt more efficiently to various root shapes, the sockets were enlarged by the use of a long cylindrical carbide bur (D&Z; Wiesbaden, Germany) and a high speed dental hand piece (Henry Schein Company; Prague, Czech Republic) under water coolant system. Samples were randomly selected from the six groups, placed into sockets, and fixed with red wax.

**Imaging**

DPR was made from each placed tooth at three horizontal angles. In the first group, the X-ray beam was perpendicular to the tooth long axis and receptor (orthogonal), the second group was imaged at 20° mesial angulation, and the third group was imaged at 20° distal angulation. The radiologic images were made using an intraoral charge-coupled device (CCD) sensor (RSV; Visiodent, St Denis, France) and the X-ray was produced by an intra-oral X-ray unit (Planmeca; Helsinki, Finland) at 70 kVp, 0.2 s and 10 mA. Visiodent imaging software (Visiodent; France) was used to edit the images.

CBCT images were taken from each tooth  using Cranex 3D (Soredex; Helsinki, Finland) at 89 kV, 6 mA and 12.6 s with 8×4 cm field of view (FOV); the voxel size was 0.2 mm. The images were processed by using OnDemand3DDental software (Soredex; Helsinki, Finland) and were reconstructed in the axial, sagittal, and coronal planes with the slice thickness and cross interval of 0.2 mm.

All the images were displayed on a 19‑inch LG Flatron monitor (E1940S; LG, Seoul, Korea) (resolution=1280×768 pixels, color depth=32 bit) in a dimly lighted room.

**Data collection**

Two blind observers (an endodontist and a maxillofacial radiologist) investigated the DPR and CBCT images independently and recorded their diagnosis on a dichotomous scale (positive or negative). Negative score was considered for the teeth with no evidence of fracture in any of the three DPRs. The other conditions were considered as positive. In CBCT images, fracture was detected in each of the three planes separately. An overall diagnosis was reported for each tooth based on images in all sections. Positive was considered if fracture was observed in at least one of the three planes of the sections; otherwise, the diagnosis was recorded as negative. The observers were allowed to change the contrast, brightness, invert, and zoom options on all images. Two involved observers were educated and calibrated in a pilot study to detect VRF in CBCT and DPR images. In addition to individual reports of each observer, a consensual diagnosis was reported for each tooth based on the diagnosis of both observers. In cases of disagreement between the two observers, they were asked to reach an agreement. 

**Data analysis**

Sensitivity (correct detection of the fractured teeth), specificity (correct detection of the non-fractured teeth), and accuracy (correct detection of non-fractured and fractured teeth) were calculated for CBCT and DPR images inspected by each observer separately in the three groups. Chi-square test was used to assess the difference between sensitivity and specificity values. Kappa index was used to assess inter-observer agreement (*p*< 0.05) and also to assess consensus of CBCT and DPR versus the gold standard in each of the three groups. The data were analyzed by using SPSS software for Windows (version 16.0; SPSS Inc.). 

## Results

Generally, CBCT had higher sensitivity, but lower specificity compared with DPR (Table 1). However, Chi-square test showed the difference to be insignificant (Table 2).

**Table 1:** The sensitivity, specificity and accuracy of CBCT and DPR in each subgroup

	**CBCT**									**DPR (with three different horizontal angles)**								
	**No canal filling**			**Gutta-percha**			**Post**			**No canal filling**			**Gutta-percha**			**Post**		
	1	**2**	**C**	**1**	**2**	**C**	**1**	**2**	**C**	**1**	**2**	**C**	**1**	**2**	**C**	**1**	**2**	**C**
Sensitivity	0.95	1.00	1.00	0.80	0.80	0.80	0.75	0.70	0.70	0.80	0.80	0.80	0.65	0.65	0.60	0.65	0.75	0.85
Specificity	0.90	0.90	0.90	0.65	0.60	0.60	0.65	0.65	0.65	0.90	0.95	0.95	0.75	0.70	0.75	0.70	0.70	0.70
Accuracy	0.92	0.95	0.97	0.72	0.70	0.75	0.70	0.67	0.67	0.85	0.87	0.87	0.70	0.67	0.67	0.67	0.72	0.77

CBCT: cone beam computed tomography; DPR: digital periapical radiography; 1: observer 1 (radiologist); 2: observer 2 (endodontist); C: consensual report of the two observers

**Table 2:** P values of the difference between sensitivity, specificity, and accuracy of the two imaging techniques in each subgroup obtained by using chi-square test.

	**No canal filling**	**Gutta-percha**	**Post**
Sensitivity	0.635	0.447	0.566
Specificity	0.548	0.168	0.736
Accuracy	0.235	0.809	0.317

The overall sensitivity and specificity of orthogonal DPR were 0.53 and 0.85 respectively. By using three angulations, the sensitivity of DPR was improved to 0.75 but the specificity was reduced to 0.80. Chi-square test showed the difference between the sensitivities were significant (*p*= 0.013); whereas, the difference between the specificities was insignificant (*p*= 0.471). Consensus of CBCT and DPR versus the gold standard was higher for teeth with no canal filling than those in the two other subgroups. Higher agreement with the gold standard was obtained for CBCT compared with DPR in no filling and gutta-percha groups. But in the post group, the kappa value showed higher agreement for DPR (Table 3).

**Table 3:** Kappa values of agreement between CBCT, DPR, and the gold standard in the three subgroups

	**CBCT**	**DPR**
No canal filling	0.900, *p*< 0.001	0.750, *p*< 0.001
Gutta-percha	0.400, *p*= 0.010	0.350, *p*=0.025
Post	0.350, *p*=0.027	0.550, *p*=0.001

Inter-observer agreement was higher for CBCT (κ= 0.882, *p*< 0.001) in comparison with DPR (κ= 0.732, *p*< 00.1)

## Discussion

Based on our results, CBCT was more sensitive but less specific compared with DPR for detection of VRF in root filled posterior teeth; however, the difference was insignificant. A systematic review has recently mentioned that *in vivo* studies reach higher values of sensitivity for CBCT rather than for DPR; which is in common with our results. However, the difference was stated to be significant. Moreover, the specificity of DPR was reported to be higher, but comparable with that of CBCT.[13] Regarding the higher radiation dose of CBCT,[14] our study supports the application of PR with three different horizontal angels in the cases suspected to VRF. In the cases with no filling material in the canal, the fracture was detected more probably and CBCT was more accurate. In the presence of gutta-percha or post in the canal, the sensitivity and specificity of both imaging modalities were decreased. 

**Figure 1:**
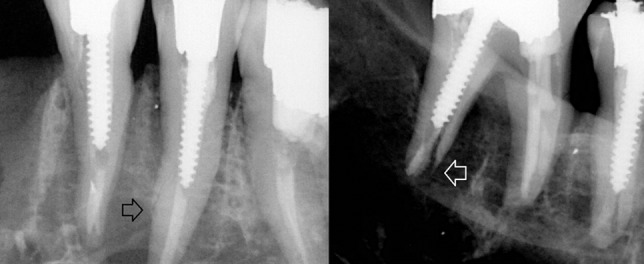
The fracture line in a root-filled and post cemented tooth is detectable (arrow) in these digital periapical radiographs.

The sensitivity of CBCT was higher in no filling and gutta-percha groups, but lower in post group. This finding indicates that the fracture is more likely to be discovered in periapical radiographies rather than in CBCT when a cemented radio-opaque post exists in the canal (Figure 1).

Consistent with the findings of Kambungton *et al*.,[9] the difference was insignificant; while, Hassan *et al.*[7]reported a significant difference between the sensitivity of these two modalities. CBCT provides a three-dimensional (3D) image of the tooth; so the fracture is more likely discoverable in a 3D image like CBCT rather than in a two-dimensional image obtained by PR. Accordingly, the sensitivity values are greater for CBCT. 

As previously demonstrated, the sensitivity of CBCT is less dependent on the orientation of the fracture line compared with PR. The sensitivity of PR increases as the percentage of buccolingual fractures increases in a sample. Considering the greater number of buccolingual fractures in our study, the sensitivity of DPR may be lower than the reported values in this study. But, since the buccolingual fractures are more common in clinical cases,[1] *in vivo* studies might report approximately similar results. As in an *in vivo* study, CBCT was shown to have higher sensitivity and lower specificity compared with DPR and the differences were insignificant.[15] The reported values of sensitivity for both CBCT and DPR by Chavda *et al.*[15] (0.27 and 0.16, respectively) were lower than those reported by Wenzel *et al.*[16] (0.87 and 0.74) and its minimum value in our study (0.70 and 0.65). This may be due to the *in vitro* nature of the two latter studies. 

**Figure 2:**
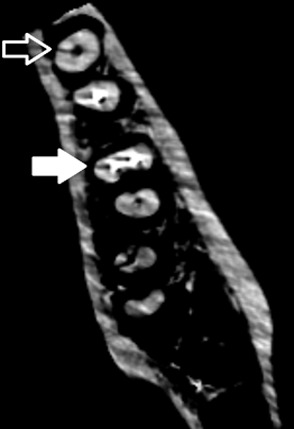
An axial section of CBCT shows a fracture in a non-filled root (the hollow arrow) and a streaking artifact mimics the fracture line in a root canal filled with gutta-percha (the solid arrow)**

Our results corroborate the previous findings that reported DPR was more specific than CBCT for VRF diagnosis in root-filled teeth.[7, 17] Inconsistent with a study that reported the significance of the difference between the specificity of the two modalities,[17] our findings confirmed the insignificance of this difference.[7] Higher DPR specificity is probably due to the two-dimensional nature of DPR. Fractures are more likely to be covered by root filling materials and superimpositions in DPR. Thus, the teeth are mostly reported as non-fractured and the number of false positives will be reduced. On the other hand, root filling materials can lead to beam hardening and produce streaking artifact in CBCT images. This artifact may mimic the fracture line and, therefore, mislead the clinician to mistake this low-density area for a root fracture.[18-19] (Figure 2)

Thus, the number of false positives increases and the specificity reduces. Lower CBCT specificity values in the presence of canal fillings in our study could be attributed to the occurred artifacts.

The sensitivity of DPR improved by using three different horizontal angels, and the specificity reduction was insignificant. Taken together, the three angulations approach (TAA) is more effective than a single orthogonal DPR (ODPR) for proper detection of VRF. Previous studies confirm this finding.[9, 20] In our investigation, the positive report for ODPR led to positive report for TAA. It is clear that the number of positive reports for TAA would be at least equal to that of ODPR. So, mathematically speaking, the sensitivity of TAA must be equal to or greater than ODPR. Significantly higher value of the sensitivity, when using three angulations, indicated that images taken at distal and mesial angles revealed some fractures that were not detected in ODPR. Hence, this study supported the idea of using different horizontal angels. The higher specificity of ODPR meant that false positive reports were more in TAA than in ODPR. Furthermore, adjacent structures may be superimposed on the images obtained at mesial and distal angulations. These superimpositions are probable to be misinterpreted as fracture. Soft and hard tissues can have a similar effect *in vivo*. Similar results were obtained by Kambungton *et al.*[9] and Wenzel *et al.*[20] who investigated 120 and 48 teeth, respectively. Although the difference was insignificant   the sample size of the present study and that of the above-mentioned studies, it may be significant in a larger sample size. Further *in vivo* studies with larger sample sizes are required. However, due to the ethical considerations, assessment of the three angulations on human would bring some limitations. 

In line with the results of a previous study,[8] the inter-observer agreement was higher for CBCT. This finding suggests that there is more agreement between different observers for CBCT than PR. Inter-observer agreement decreased in the case of root-filled teeth. For CBCT images, the reduction was more in post cemented group. It was in agreement with what Naves *et al.*[21] reported. The images in the current study were reviewed by only two observers; while more observers are needed to conclude more confidently.

The tooth missing structure should be restored after root canal therapy; metal restorations are commonly used to achieve this purpose in posterior teeth.[22] Similar to the root filling materials, these restorations can cause streaking artifact in CBCT images and may influence its diagnostic value. To our knowledge, none of the previous *in vitro* studies restored the teeth with metal restorations. To achieve more similarity to the real clinical conditions in this study, after root canal therapy, the access cavity was filled with amalgam as a dense metal restoration. Nonetheless, we did not evaluate the effect of the coronal filling on VRF diagnosis. Further studies might be needed to find out its possible effects.

The present study did not measure the width of the fracture, either. Wider cracks are significantly more detectable in CBCT.[14] Obtaining different values of sensitivity and specificity in studies with no control over the fracture width might be ascribed to different methodologies which resulted in different crack widths.[17] Moreover, the lower values reported in an *in vivo* study compared with *in vitro* investigations may be due to the greater width of an artificially induced fracture.[15] Although the fracture width is a confounding variable over which we had no control, it must be considered that it is difficult to measure the fracture width in many teeth. 

Due to dedicated traits and application of different CBCT devices, the results of this study can be attributed to the employed system. Using other machines may yield different results. 

## Conclusion

There was no statistically significant difference between the CBCT and a set of three PRs prepared at different horizontal angels (20°mesial, 0°, and 20° distal) for *in vitro* VRF detection in posterior teeth. 
